# Current challenges in software solutions for mass spectrometry-based quantitative proteomics

**DOI:** 10.1007/s00726-012-1289-8

**Published:** 2012-07-22

**Authors:** Salvatore Cappadona, Peter R. Baker, Pedro R. Cutillas, Albert J. R. Heck, Bas van Breukelen

**Affiliations:** 1 Biomolecular Mass Spectrometry and Proteomics Group, Bijvoet Centre for Biomolecular Research and Utrecht Institute for Pharmaceutical Sciences, Utrecht University, Padualaan 8, 3584 CH Utrecht, The Netherlands; 2 Netherlands Proteomics Centre, Padualaan 8, 3584 CH Utrecht, The Netherlands; 3 Department of Pharmaceutical Chemistry, Mass Spectrometry Facility, University of California San Francisco, San Francisco, USA; 4 Analytical Signalling Group, Centre for Cell Signalling, Barts Cancer Institute, Barts and The London School of Medicine and Dentistry, Queen Mary University of London, Charterhouse Square, London, EC1M 6BQ UK; 5 Netherlands Bioinformatics Centre, Padualaan 8, 3584 CH Utrecht, The Netherlands

**Keywords:** LC–MS, Quantitative proteomics, Quantification software, Stable isotope labeling, Label-free

## Abstract

Mass spectrometry-based proteomics has evolved as a high-throughput research field over the past decade. Significant advances in instrumentation, and the ability to produce huge volumes of data, have emphasized the need for adequate data analysis tools, which are nowadays often considered the main bottleneck for proteomics development. This review highlights important issues that directly impact the effectiveness of proteomic quantitation and educates software developers and end-users on available computational solutions to correct for the occurrence of these factors. Potential sources of errors specific for stable isotope-based methods or label-free approaches are explicitly outlined. The overall aim focuses on a generic proteomic workflow.

## Introduction

Until the last decade, proteomics was essentially a descriptive discipline, but the fast development of mass spectrometry-based proteomic technologies, and the accessibility of powerful data analysis tools, has increasingly boosted the transition of proteomic analysis from qualitative to quantitative (Ong and Mann 2005), with a strong impact on biological interpretation of protein functions (Cox and Mann 2011).

Several strategies for protein quantitation are possible, including *gel*-*based* and *mass spectrometry*-*based* methods.

*Gel*-*based* quantitation methods rely on relative abundance measurement of gel bands (in 1D SDS-PAGE gels) or gel spots (in 2D gels) across the samples being compared (Weiss and Görg 2009). This technology is able to separate more than 10,000 spots on a single electrophoretic run, but suffers from poor gel reproducibility and frequent co-migration of multiple proteins under individual spots. An important advance in gel-based quantitation occurred when the DIGE technology (Unlü et al. 1997) allowed the use of fluorescent dyes to label and separate different protein samples on the same gel, thus effectively solving the reproducibility issue. Furthermore, the protein co-migration issue is currently addressed by spot excision and further quantitative analysis by mass spectrometry.

*Mass spectrometry* (*MS*)-*based* quantitation methods rely on the linearity of MS ion signal versus molecular concentration (Purves et al. 1998), initially confirmed for protein abundances by Chelius and Bondarenko (2002). Due to better sensitivity of current MS platforms for low molecular weight molecules, these methods have actually evolved in a somewhat counterintuitive peptide-centric way, based on the assumption that proteins in the original sample can be identified and quantified by means of MS-mediated identification and quantification of their constituent proteolytic peptides (Duncan et al. 2010). This reverse engineering approach is often referred to as *shotgun* or *bottom*-*up* proteomics, to distinguish it from the more intuitive measurement of intact proteins, known as *top*-*down* proteomics (Collier et al. 2008; Kellie et al. 2010; Waanders et al. 2007).

The main methods devised in recent years for MS-based protein quantitation have already been extensively reviewed (Bantscheff et al. 2007; Becker and Bern 2011; Ong and Mann 2005; Schulze and Usadel 2010; Yan and Chen 2005) along with their advantages and disadvantages (Elliott et al. 2009; Mann 2009) and within specific applications and contexts (Cox and Mann 2011; Macek et al. 2009; Simpson et al. 2009; Timms and Cutillas 2010). Broadly speaking, they can be classified as *stable*-*isotope*-*labeling* (Julka and Regnier 2004; Leitner and Lindner 2004), based on introducing a mass tag into proteins or peptides, either metabolically, enzymatically or by chemical means; and *label*-*free* approaches (America and Cordewener 2008; Lundgren et al. 2010; Neilson et al. 2011; Podwojski et al. 2010; Zhu et al. 2010), which correlate the ion current signal of intact proteolytic peptides or the number of peptide spectral match counts directly with the absolute protein quantity. Reproducibility (Kim et al. 2007) and comparison (Hendrickson et al. 2006) of the various relative quantification strategies have also been widely assessed.

A clear message emerging from recent proteomics literature is the necessity for robust software tools for data processing, whose development is lagging behind the substantial advances in instrumentation and methodologies. Current software packages for performing quantitative proteomics have been recently reviewed (Codrea et al. 2007; Jacob 2010; Matthiesen 2007; Mueller et al. 2008), and effective metrics for software comparison have been proposed for both labeled (Colaert et al. 2011) and label-free (Sandin et al. 2011) approaches.

Building on this extensive literature, this review gives an overview of the critical factors contributing to incorrect measurements and further elaborates on available strategies to detect quantification errors and possibly correct them.

The remainder of this section will summarize the main aspects of labeled and label-free approaches. The following section will then summarize a checklist of ten current challenges to consider when evaluating software solutions for quantitative proteomics. The description will follow a typical quantitative proteomics workflow, starting from pre-processing and feature detection, moving to peptide identification and quantification, then continuing with protein inference and quantification and concluding with a section on post-analysis statistical methods. Although the major part of the discussion focuses on stable isotope-based quantification, distinctions and caveats for label-free approaches will be explicitly raised when necessary.

## Generic LC–MS quantitative proteomics workflow

In a typical proteomics experiment (Aebersold and Mann 2003), proteins are digested to peptides by a site-specific enzymatic protease, such as trypsin. The resulting peptides are then separated by liquid chromatography (LC), converted to gas phase ions and analyzed by MS. The mass spectrometer scans the whole mass range and produces high-resolution MS spectra (a mass resolution of 60,000 full width at half maximum, FWHM, is routine on current instruments).

The acquisition software then automatically selects a preset number of peptides for fragmentation and for further analysis by so-called tandem mass spectrometry (MS/MS). Current instruments allow the acquisition of one MS *survey scan* every few seconds, each followed by tens of *data*-*dependent* MS/MS spectra after each MS spectrum. The resulting MS/MS spectra are finally compared either to theoretical fragmentation spectra generated from a protein sequence database or to spectral libraries, in order to retrieve the corresponding peptide sequences (Steen and Mann 2004). Current computational tools allow the unambiguous identification of more than half of all tandem mass spectra (Cox and Mann 2008), typically verified by stringent community requirements (Bradshaw et al. 2006) and robust techniques for determining false positives (Elias and Gygi 2007).

**Fig. 1:**
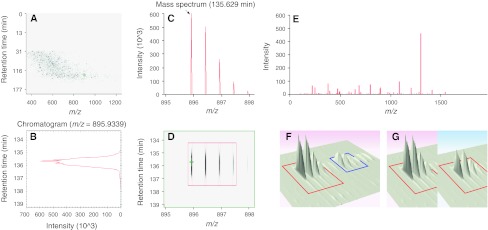
LC–MS signals. **a** The ion intensity map gives a bird-eye view of the whole LC–MS experiment. Highlighted in *green* is a peptide feature magnified in *panel d*. **b** Extracted ion chromatogram (XIC) of the monoisotopic *peak* of the selected peptide ion. The signal shows the ion intensity as a function of the elution time. The area under the curve (AUC) represents the total signal of the monoisotopic *peak*. **c** Mass spectrum of the selected peptide ion at maximum chromatographic intensity. The *m*/*z* difference of 0.5 Th between contiguous isotopic *peaks* allows deriving a charge state of 2. The *arrow* indicates the monoisotopic *peak*. **d** Ion intensity map of the peptide ion of interest. The *green cross* indicates the precursor ion selected for fragmentation. **e** Tandem mass spectrum of the monoisotopic peak of the selected peptide ion, highlighted by a *green cross* in *panel d*. The mass difference between selected *peaks* allows deriving the amino acids sequence. **f** For stable isotope-based quantification peptides from two different samples are detected in the same LC–MS run at a characteristic mass difference. **g** For label-free quantification corresponding peptides from two different samples are detected at the same mass and similar retention time in two different LC–MS runs

All signals produced by the mass spectrometer and available for further processing are sketched in Fig. 1, which will be used as a reference throughout this paper.

Consecutive protein identification is inferred from peptide data. One or two protein-specific peptides are typically enough to confirm the presence of a protein within the sample, but higher sequence coverage is required to distinguish isoforms and post-translational modifications (PTMs).

Most biological studies increasingly require further quantitative inputs. It is worth noting that in proteomics the term ‘quantification’ is used quite loosely, as most biological questions actually imply only *relative* comparison of protein amounts in different samples or states, or in response to experimental perturbations. Since mass spectrometry is not well suited for measuring absolute amounts, *absolute* quantification, if needed, is usually determined by comparison to an internal isotope-free (Steen et al. 2005) or isotope-labeled (Gerber et al. 2003) standard.

The two main approaches to make MS-based proteomics quantitative, stable-isotope-labeling and label-free will be summarized in the next two sections.

## Stable isotope-based quantitative proteomics

The most popular approaches for relative quantification are based on labeling proteins or peptides in at least one of the compared samples with compounds enriched in stable heavy isotopes of hydrogen, carbon, nitrogen or oxygen (Heck and Krijgsveld 2004). These approaches exploit the fact that labeled molecules behave almost identically during chromatographic separation, ionization and in the mass analyzers; yet, they can be easily distinguished from their unlabeled counterparts thanks to the mass shift endowed by the heavy isotopes (Fig. 1f).

Many different methods for quantitative proteomics based on isotope labeling have been described, often classified by the way the labels are introduced into peptides or proteins. In *metabolic labeling*, the label is introduced to the whole cell or organism in vivo, through the growth medium, while in *chemical labeling* the label is added to proteins or tryptic peptides through chemical derivatization or enzymatic modification in vitro, after sample collection. An important advantage of metabolic incorporation is that the labels are present in the living cells. This means that the samples from the different quantification states can be combined directly after cell lysis, thus reducing sample processing variability and allowing higher quantitative accuracy. Conversely, the main advantage of chemical labeling is its applicability to virtually any type of sample.

A popular *metabolic labeling* method is stable isotope labeling by amino acids in cell culture (SILAC) (Ong and Mann 2005). In SILAC, essential amino acids such as arginine and lysine are provided in ‘light’ or ‘heavy’ forms to the two cell populations and are incorporated into each protein after a few cell doublings, leading to a well-defined mass difference. A drawback of SILAC is that its application is limited to amino acids auxotrophs, in order to make sure that only labeled amino acids are incorporated into proteins. An established alternative technique, which allows the complete labeling of virtually all amino acids in expressed proteins of both prototrophs and auxotrophs, is ^15^N-labeling (Oda et al. 1999), through the metabolic incorporation of inexpensive labeled ammonium salts. The advantages of this technique come at the price of a more difficult detection of the peptide pairs, because the mass difference depends on the amino acid composition.

*Chemical labeling* makes use of externally introduced isotopic or isobaric reagents. Examples of the first category include dimethyl labeling (Boersema et al. 2009; Kovanich et al. 2012) and isotope-coded affinity tag (ICAT) (Gygi et al. 1999). Isobaric mass tagging, exemplified by isobaric tag for relative and absolute quantitation (iTRAQ) (Ross et al. 2004) and tandem mass tags (TMT), (Thompson et al. 2003), differs from the methods described above in that labeled peptides have almost exactly the same mass and are thus indistinguishable in the survey spectra. In this case, the different mass tags separate only upon fragmentation and quantitation relies on the intensity ratios of so-called reporter ions in the fragment spectra. Note that tandem MS identifications have been recently reported also in the absence of detectable precursor signals (Panchaud et al. 2009), suggesting that isobaric methods may be more sensitive than isotopic ones. The last approach for differential quantification by chemical derivatization is *enzymatic labeling*, exemplified by ^16^O/^18^O labeling (Mirgorodskaya et al. 2000), where the mass tag is introduced in the peptide chain by performing proteolytic digestion in the presence of heavy water.

For a deeper assessment of the principles of isotope labeling in proteomics the reader is referred to more comprehensive reviews (Heck and Krijgsveld 2004; Timms and Cutillas 2010). For the purpose of this review we highlight incomplete labeling, chromatographic shifts and isotopic overlaps as the main issues related to stable-isotope labeling that will be further discussed in this manuscript.

## Label-free quantitative proteomics

Although protein relative quantification using labeling strategies has been successfully used in many studies, these techniques are strongly limited by the number of samples that can be compared. Consequently, there is currently considerable interest in the proteomics community for quantitative MS methods that do not require isotope labels and that rely on direct comparison of peptide signals across different experiments (Fig. 1g). These so-called *label*-*free* methods offer two main advantages that are particularly suited for studies that require statistical analysis of technical and biological replicates, namely simpler sample preparation and direct comparison of multiple samples.

In its simplest form, the number of peptide fragmentation events is taken as an estimate of the amount of protein (Liu et al. 2004). This spectral counting technique has been used to provide a semi-quantitative measure of protein abundance (Ishihama et al. 2005; Lu et al. 2007; Old et al. 2005) but has been found to often give irreproducible data (Griffin et al. 2010). Taking into account the intensities of MS/MS spectra in addition to the number of such spectra matched to proteins has been reported to increase the accuracy of the measurement (Sardiu and Washburn 2010), but this has not been confirmed by other groups. The advent of high-resolution mass spectrometry has made it easier to measure and compare the actual signals of peptide ions in survey scans. In contrast to spectral counting techniques, label-free methods based on the use of ion currents were found to provide a level of accuracy comparable to labeling approaches (Casado and Cutillas 2011; Chelius and Bondarenko 2002; Cutillas and Vanhaesebroeck 2007). Issues specific to label-free approaches based on ion currents will be explicitly highlighted below. The most common readouts are extracted ion chromatograms (XIC) of the parent ion, although other readouts of peptide abundance can be used, such as monitoring fragment ion intensities by selected/multiple reaction monitoring (SRM/MRM, Lange et al. 2008).

## Software assessment checklist: 10 current challenges

The most important step of a proteomic workflow is undoubtedly feature detection. Since it is difficult to find agreement on the definition of LC–MS peaks and features, in this article we will term a *peptide feature* as the whole profile generated by the elution of a peptide in an LC–MS map (Fig. 1d); and a *peptide peak* as each of the isotopic components of a peptide feature, like the monoisotopic peak pointed out in Fig. 1c.

The detection and quantification of a peptide feature from a raw LC–MS map is a complex procedure that relies on measurement of the mass, charge and abundance of its peaks, detection of the monoisotopic peak, deisotoping and deconvolution from contaminant peaks. For effective feature detection, it is good practice to first perform pre-processing steps, such as data reduction, noise filtering, background subtraction, mass calibration and retention time alignment, in order to clean up the data. The potential pre-processing requirements vary somewhat with the type of instrument used and a full description is certainly beyond the scope of this article. The most relevant steps for our purposes will be covered in the next sections, while we recommend recent reviews by Zhang et al. (2009) and Matthiesen et al. (2011) for more details on this topic.

## Challenge 1: software usability

In order to become adopted by a large audience, software tools need to be intuitive and easy to use. While writing this manuscript, many of the available quantitation software tools were evaluated to assess whether they tackled the issues enumerated below. Strikingly, many putatively good tools, including tools that addressed many issues related to accurate quantification, were difficult to use. Most of the time, this was due to lack of appropriate documentation or to a poor graphical user interface.

From the *end*-*user* point of view, the most relevant issues perceived when evaluating a new quantification tool are mostly related to: (1) ease of installation. Is the tool at hand easy to install, or does it require expert knowledge? For example, can you use an installer, or does it require manual compiling from the source code? (2) Presence of documentation or tutorials, which help in perceiving the software as ‘easy to use’. (3) Presence of a graphical user interface. (4) Presence of interactive feedback during data processing, to allow for adjustments and ad hoc decision making. (5) Presence of interactive feedback during the quantification process to allow for manual validation of the quantification results or visual assessment of what went wrong in case of no results. (6) Presence of a mailing list, for update notifications, discussion about problems and direct help from the software developers. (7) Storage and sharing of user data and results.

From a *bioinformatics developer* perspective, relevant caveats when designing a new software tool should include: (1) flexibility, i.e., how well does the software follow current standards and/or does it handle multiple vendor formats? (2) Modularity, i.e., can the software be easily integrated into existing pipelines or workflow management tools (e.g., Taverna.^1^) (3) Portability, i.e., can the software run on different hardware platforms? (4) Documentation. (5) Distribution terms: freeware, shareware or commercial? Open source or closed source? Web based? (6) Scaling and parallel processing, i.e., are multithreading, multiprocessing or grid-based processing possible? (7) Batch processing, i.e., is it possible to run large batches of files in a single instance and without manual intervention? For a detailed review the reader is referred to Codrea et al. (2007), where several LC–MS processing tools are extensively evaluated based on their software usability.

## Challenge 2: data reduction

Several software packages allow storing and direct handling of the acquired raw data files, intended as the proprietary binary output provided by the instrument. Protein Prospector (Chalkley et al. 2005), for instance, can accommodate whole laboratory repositories (Lynn et al. 2005) and retrieves all relevant data required for quantification directly from the original files. However, the raw files are usually considered too big to be handled directly by downstream analysis algorithms. Furthermore, converting them to standard formats, like mzML (Deutsch 2008), only worsens the situation. For this reason, data reduction is often one of the first steps in data processing, so that only the necessary data are retained for further analysis.

### MS data reduction

Listgarten and Emili (2005) point out that a matrix representing the whole LC–MS map is all that is necessary for further data processing. Each cell in the matrix represents the ion abundance at a given combination of retention time (RT) and mass-over-charge (*m*/*z*) ratios. Since digital signal processing requires regular sampling, the matrix formation is necessarily related to re-sampling or binning data in both dimensions—in time, because MS spectra might not be taken at regular intervals, and in mass, because most instruments apply a nonlinear transformation to the acquired data to determine the *m*/*z* values.

In general, data reduction is intended as reducing the raw data to a more manageable set of peaks (Martens 2011). A basic step toward size reduction can be obtained by centroiding the MS spectra, a procedure by which a single peak is retained to represent the center of the *m*/*z* ion distribution measured by the instrument detector. A further reduction can be obtained by reducing each peptide feature to a simple triplet <*m*/*z*, RT, I>, representing the exact mass, retention time and intensity of its monoisotopic peak. The set of all triplets from an LC–MS map is all that is necessary to perform data mining by established techniques drawn from signal processing, statistics and machine learning. However, we strongly suggest postponing all data reduction steps that go beyond mere signal processing until after gathering more information from downstream analysis. In fact, performing these advanced steps before disentangling the peptide features from noise and contaminants, and before aligning them and normalizing them, can negatively affect quantification accuracy.

### MS/MS data reduction

MS/MS spectra are usually acquired in centroid mode and are thus much smaller in size than survey scans. MS/MS data reduction methods, therefore, are not aimed at size reduction, but rather at filtering spectra to increase the efficiency and effectiveness of subsequent database or library search algorithms. If the MS/MS spectra are going to be used for isobaric quantification, it is also important that any reduction method does not distort the reporter ions. MS/MS data reduction strategies mainly focus on the following areas: (1) Pre-processing to centroid peaks, filter out noise, deconvolute multiply charged ions to the *m*/*z* of the corresponding 1+ charge state, and deal with isotope clusters. (2) Detection and clustering of multiple redundant spectra of the same peptide (Beer et al. 2004; Tabb et al. 2005). From the point of view of quantification, clustering algorithms may be useful for the detection of weaker peptides. (3) Detection of spectra of multiple co-eluting peptides (Bern et al. 2010; Houel et al. 2010) which can seriously harm identification and quantification. (4) Elimination of low-quality spectra (Flikka et al. 2006; Junqueira et al. 2008). (5) Reassignment of precursor charge and *m*/*z* (Mayampurath et al. 2008; Shinkawa et al. 2009).

It should be noted that the increasing popularity of data-dependent decision tree logics for regulated combination of fragmentation techniques (Frese et al. 2011; Swaney et al. 2008) is triggering the development of customized pre-processing algorithms. ETD spectra, for instance, require a bespoke strategy because of hydrogen transfer and the presence of neutral loss ions (Baker et al. 2010; Good et al. 2009). Similarly, HCD spectra require tailored steps for deisotoping, deconvolution and even rescoring of the high mass accuracy spectra (Savitski et al. 2010).

## Challenge 3: feature detection

As shown in Fig. 1, a peptide feature is composed of multiple peaks at different *m*/*z* locations, a phenomenon known as isotope dispersion. Since proper quantification relies on accurate feature detection, recognizing the isotopic pattern and cleaning it up from all interferences are paramount for abundance measurement and all subsequent analyses.

### Deisotoping (and abundance measurements)

Several methods have been proposed for measuring the abundance of a peptide feature. The easiest quantity to be measured is the summed area of all isotopic peaks in a given scan (Fig. 1c), usually the survey scan or the scan where the elution profile reaches its maximum intensity. The single scan areas can also be averaged or summed over the whole peptide elution time, the latter of which gives an estimate of the feature volume (Cox and Mann 2008; MacCoss et al. 2003). As using the whole isotope profile makes the precursor peak more vulnerable to contamination from co-eluting isobaric compounds, many software tools, such as XPRESS (Han et al. 2001) and MSQuant (Mortensen et al. 2010), only calculate the abundance of the monoisotopic peak, although this is known to reduce the sensitivity at higher masses (e.g., the monoisotopic peak is 5 % of the total abundance at 5,000 Da).

When measuring the areas of the peaks for quantification, most tools only consider features in precursor mass spectra near a fragmentation event. In this case the composition of the peptide will generally have been determined by a database search and it is thus possible to calculate the theoretical isotope distribution and compare it with the experimentally measured one. A goodness of fit metric, such as Pearson’s Chi-squared error between the theoretical and experimental distributions could then be used to flag potentially suspect measurements (Valkenborg et al. 2007).

Recent tools, such as MaxQuant (Cox and Mann 2008) and PVIEW (Khan et al. 2009) adopt the opposite approach and anticipate feature detection in parent mass spectra, to use all available constraints for driving a database search. In these cases, where the peptide sequence is unknown, the *averagine* model can be used to estimate peptide isotopic distributions, based on the assumption that the dependence of mass is a good approximation to the dependence on sequence (Senko et al. 1995). We are not aware of any current tools that double-check the isotopic pattern after the peptide assignment.

### Isobaric interference from isotopic clusters

**Fig. 2:**
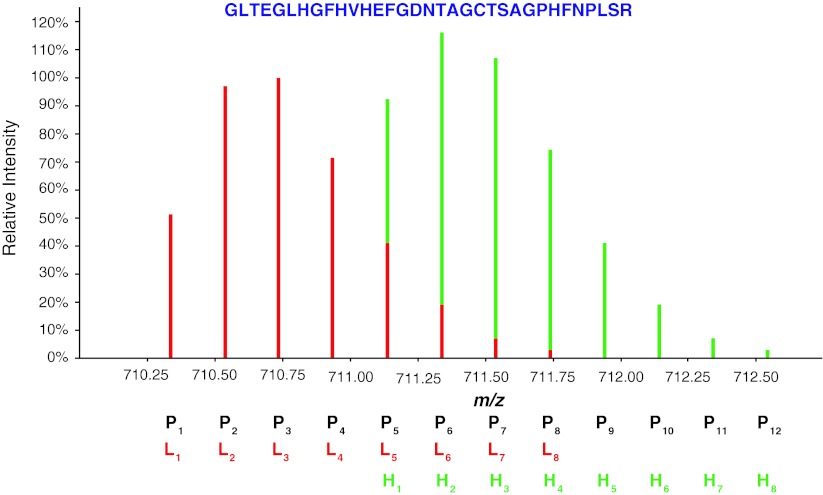
Overlapping isotopic clusters. Isotopic distribution of the dimethyl labeled peptide GLTEGLHGFHVHEFGDNTAGCTSAGPHFNPLSR. The mass shift of the two isotopologues is smaller than their isotopic envelope, resulting in the overlap of the fifth and consecutive *peaks* of the light peptide on the monoisotopic and consecutive *peaks* of the heavy one. In this example, the two peptides are equally abundant, but a quantification strategy that evaluates peptide ratios based on their monoisotopic *peaks* would largely overestimate the heavy peptide

For the labeled pair shown in Fig. 1f, the two isotope profiles are distinct. In this case the quantification ratio can be easily calculated as the abundance ratio of the two features over their total elution time. Although potentially straightforward, this quantitative strategy can be hampered by the overlap of isotopic clusters of light and heavy peaks, which occurs whenever the mass shift between the peptide pairs is smaller than their isotopic envelope. The phenomenon is significantly apparent for heavier peptides, which have a larger number of isotopic peaks and thus usually show a trend toward an overestimation of the heavier isotopologues (Fig. 2).

**Fig. 3:**
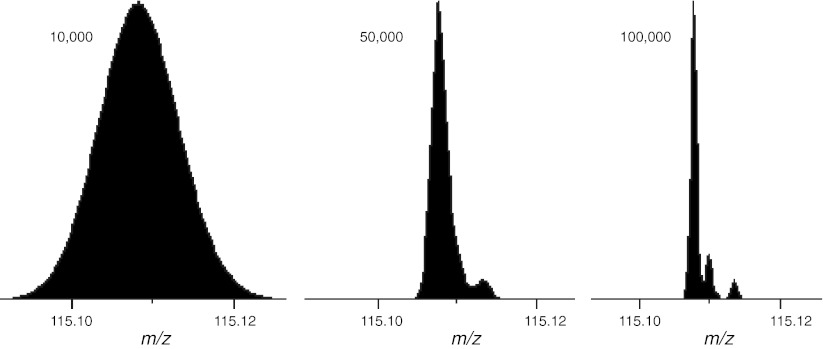
Theoretical iTRAQ data in the region around the 115 reporter ion at different resolutions. The model assumes 95 % purity for ^13^C and ^15^N and a 1:1:1:1 mixing ratio. The three peaks seen at a resolution of 100,000 FWHM are (from *left* to *right*) the monoisotopic 115 *peak*, the *peak* from the partial enrichment in the 116 reporter ion and the first isotope peak from the 114 reporter ion. Analysis performed using Protein Prospector (Chalkley et al. 2005)

The general mathematical strategy to correct for the overlap of isotopic clusters consists of subtracting the contribution of the interfering isotopes of the light form of a labeled pair from the peaks of the heavy form. Meija and Caruso (2004) discuss three different methods for deconvoluting isobaric interferences: one in the intensity domain and two in the mass domain. *Deconvolution in the intensity domain* reconstructs the observed isotope pattern by superimposition of the isotope profiles of the overlapping species and adjusts the quantitative information by a least square optimization of the pattern intensities. This method necessitates solving a series of simultaneous equations using, for example, Cramer’s rule to obtain the component intensities from the measured ones. Because of the different mass defects of the elements, the masses of the isotope peaks of the lower mass component may not be exactly the same as those of the higher mass component they overlap with, resulting in a broadening of the signal in the mass domain, especially evident at low resolutions (Fig. 3). If the presence of isobaric interference is recognized, signal deconvolution of the isotopic components in the mass domain can be more appropriate. *Signal peak shape analysis* assumes that the measured signal is made up of the sum of two or more peaks of known shape, often Gaussian or Lorentzian. The parameters of the peak functions have a direct relationship to the physical properties of the measured signal, such as resolution (peak width), mass (peak position) and relative amount of the interfering species (peak area). Curve fitting can be performed by nonlinear least squares and minimized by the Levenberg–Marquardt algorithm (Press et al. 1988). *Mass shift analysis* relies on the fact that peak centroid masses are affected in the presence of isobaric interferences. For instance, when the peak width is larger than the mass difference of the unresolved isobars, the observed peak centroid mass will be approximately the weighted average of the isobar masses.

Although isobaric inference affects most isotopic labeling techniques, common quantification software still largely disregards the issue. At this moment, only a few correction tools have been proposed for isotopic deconvolution and typically they address only one specific isotopic labeling. IEMM (Dasari et al. 2009), for instance, proposes a method to overcome overlapping in ^18^O labeling, where the isotopic peaks are shifted by 2 and 4 Da. Q3 (Faca et al. 2006) predicts the isotopic distribution for acrylamide labeling, where the shift is a multiple of 3 Da, depending on the number of cysteines present in the peptides. More recently, we proposed a post-processing script to resolve overlapping peaks occurring with a 4 Da shift when dimethyl labeling is used (Cappadona et al. 2011).

The overlap of isotopic clusters also affects tandem MS-based quantification, because the isotope distributions of the lower mass reporter ions can overlap with those of the higher mass ones. Shadforth et al. (2005) have described i-Tracker, an effective implementation of intensity domain deconvolution for 4-plex iTRAQ labeling.

### Isobaric interference from co-eluting peptides

For *survey scan*-based quantitative methods, feature detection can be affected by the presence of co-eluting and nearly isobaric peptides, originating either from sample proteins or from protein contaminants. In the first case, the previously mentioned mass domain deconvolution may be used to obtain the intensities of the individual components, assuming that all the co-eluting peptides can be identified. However, it should be taken into account that such processed results will be inherently inaccurate, because of potential ion suppression from the co-eluting compound (Annesley 2003). This type of processing is becoming less necessary for high-resolution mass spectrometers, which are more likely to be able to resolve co-eluting components.

The best way to deal with peptides originating from protein contaminants is to identify them and exclude them from further analysis. Walther and Mann (2011) recently proposed a supplementary contaminant database with 255 entries. A comprehensive, but not exhaustive, list was also made available as a supplementary spreadsheet to Keller et al. (2008). Another useful resource is provided by the cRAP project, which is maintained by the Global Proteome Machine Organization. This is a list of proteins, downloadable as a FASTA database,^2^ that are often found in proteomics experiments by accident or by contamination. The database contains laboratory proteins, such as serum albumin, contact proteins, such as keratins, molecular weight standards, such as horse heart cytochrome, standard mixtures, such as the ISB Standard Protein Mix Database (Klimek et al. 2008) and common viral contaminants.

For *tandem MS*-based quantification, the contribution of co-eluting peptides depends on the size of the isolation window of the peptides chosen for fragmentation. All ions present in this window, which is typically 1–2 Th (depending on the instrument), can contribute to the signal of the reporter ions. As a result, it is not always clear to what extent quantification is contributed to by the peptide of interest or by co-eluting peptides. This can sometimes lead to a large underestimation of true changes, especially for very weak peptide signals (Ow et al. 2009). Bantscheff et al. (2008) have thoroughly investigated this problem for iTRAQ labeling and concluded that the measured fold change is increasingly deviating from the expected ratio at broader isolation widths, thus indicating that the presence of co-eluting peptides significantly affects the reporter intensities. Unfortunately, shrinking the isolation width is not always a viable solution, as it results in a significant loss of sensitivity.

Although tandem MS quantification techniques are designed to use fragmentation ‘quiet zones’ (Pappin 2004), peaks from peptide fragmentation can occasionally occur in these regions of the spectra. Table 1 lists some of the known contaminants for iTRAQ reagents. A well-known one for 8-plex iTRAQ is the first isotope peak of the phenylalanine immonium ion at 121.0839 Da (Ow et al. 2009). Another contaminant has been observed at 116.07 Da by Wolf-Yadlin et al. (2007) and, although described in other publications (e.g., Kuzyk et al. 2009), it has not yet been identified. These contaminants may be resolvable from the iTRAQ peaks with high-resolution mass spectrometers, or subtracted by one of the isobaric deconvolution methods discussed earlier.

**Table 1:** Contaminating peaks in the iTRAQ region of a tandem MS spectrum

Amino acids in peptide	Ion type	Mass (Da)
N-terminal AA	a_2_	115.0866
C-terminal P	y_1_	116.0706
C-terminal I or L	z_1_	116.0832
C-terminal N	z_1_	117.0420
N-terminal GS	a_2_	117.0659
Amidated C-terminal with C-terminal V	y_1_	117.1022
C-terminal D	z_1_	118.0261
F	Immonium (1st isotope peak)	121.0839

### Satellite peaks from partial isotope enrichment

‘Enrichment’ can be defined as the total percentage of stable isotopes in a protein. Two separate phenomena can contribute to the total degree of protein enrichment: the purity of the stable isotope obtained from the supplier and the degree of incorporation of the isotopes into proteins.

**Fig. 4:**
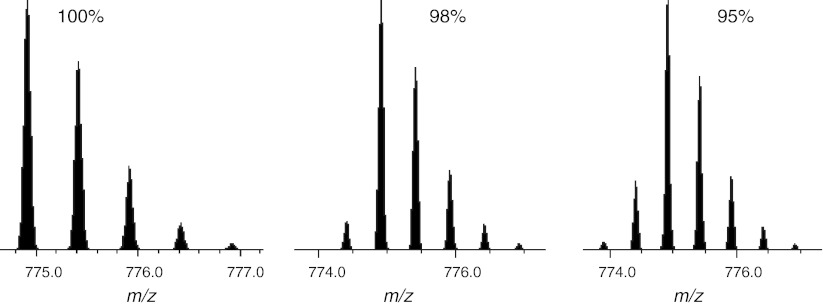
The effect of partial isotope enrichment on a labeled peptide. The *three plots* show the theoretical isotope profiles of the peptide acetyl-AAGVEAAAEVAATEIK [Label ^13^C(6)] at purities of 100, 98 and 95 %. The monoisotopic *peak* is the largest *peak* in the isotope profile and any peaks to its left are caused by partial enrichment. Analysis performed using Protein Prospector (Chalkley et al. 2005)

The first factor is a very common and almost inevitable cause of partial isotope enrichment, because commercial sources only guarantee the purity of isotope enrichment to between 95 and 98 % (although in practice 99 % is fairly common). A purity of less than 100 % will result in one or more *satellite peaks* to the left of the monoisotopic peak of any labeled peptide (Fig. 4) or tandem MS reporter ion. For this reason, tandem MS reagents always come with a data sheet indicating the percentage of each reporter ion that differs by −2, −1, +1 and +2 Da from its reporter mass, the positive offsets corresponding to the isotope peaks and the negative offsets corresponding to the satellite peaks.

The second factor that can contribute to satellite peaks originates from peptides where not all available residues in the heavily labeled sample have been labeled. In metabolic labeling, this can occur if the cells have not been grown for a sufficient number of cell doublings (Ong et al. 2002; Waanders et al. 2007), but the issue is also present with chemical labeling strategies.

In both cases, the presence of satellite peaks potentially affects both peptide identification and quantification. Identification is clearly affected because such a peak can be mistakenly considered as the monoisotopic one, resulting in incorrect mass assignment. Quantification is affected if the abundances of the satellite peaks are not added to the total peptide abundance, resulting in an artificial underestimation of the heavy peptides.

The principle of correcting for satellite peaks is the same as that for correcting for overlapping isotope profiles, if the theoretical isotope distribution is adjusted to account for partial enrichment. When modeling the isotope distribution of an enriched elemental formula, say C_48_, H_90_, N_15_, O_25_, ^13^C_6_, the ^13^C can be considered as a separate element with 100 % abundance at mass 13.003354838 (Audi and Wapstra 1995). If the enrichment is 95 % this needs to be adjusted to reflect the fact that we now have 95 % ^13^C and 5 % standard ^12^C (Boone et al. 1970).

Gouw et al. (2008) investigated the influence of ^15^N partial enrichment on the number of identifications and errors in quantification. They also described a simple correction strategy applicable to any type of labeling experiment.

### Satellite peaks from proline conversion

The use of heavy arginine as a SILAC label has been found to result in the partial labeling of proline in certain cell lines. The consequence of this is the occurrence of one or more satellite peaks depending on the number of proline residues in the peptide. For example, (^13^C_6_, ^15^N_4_)-arginine will become (^13^C_5_, ^15^N_1_)-proline, giving a mass shift of 6 Da. A peptide with two prolines will thus potentially have satellite peaks at 6 and 12 Da from the heavy peak. To correct for this, the intensities of any additional peaks need to be added to those of the heavy isotope peak before calculating the quantification ratio. Van Hoof et al. (2007) have discussed this problem in detail.

### Detector saturation

Another factor impacting the accuracy and dynamic range of quantification is saturation of the mass spectrometric detection system. Detector saturation is more often observed for Q-ToF and MALDI instruments than ion traps, as for ion traps the number of ions before detection can be controlled (Belov et al. 2003). Saturation effects are generally only a problem for survey scan-based quantification and are rarely encountered for tandem MS-based methods. If saturation occurs, the natural isotope intensity distribution is distorted, resulting in false quantitative readings. Processing software can detect the problem by comparing the measured distribution with the theoretical one for the most intense data in the data set. To correct for this, the ratios could be calculated either from the unsaturated parts of the isotope profile or using data from an unsaturated time interval in the LC–MS run.

## Challenge 4: noise rejection

We can define noise as any perturbation that hampers the detection of the peptide signal. In a typical MS experiment, there are three main sources of perturbations: random noise, chemical noise and contaminants.

*Random noise* is generally represented by small spikes, uniformly distributed in both mass and chromatographic domains. It is mainly of electrical origin and occupies the higher-end of the frequency spectrum. This kind of noise can be effectively removed by simple smoothing approaches, applied either in the LC or in the MS domain. The rational that motivates this choice is that a smooth behavior is essential for peak detection, in order to avoid picking of local maxima that are just the results of random fluctuations. Various techniques have been developed for smoothing MS spectra, including moving average, smoothing splines, wavelet smoothing and kernel methods, such as the Gaussian and the Savitzky-Golay smoothers (Hastie et al. 2009). Smoothing along the LC time axis has been performed by Savitzky-Golay, median filters and matched filtration (Andreev et al. 2003).

**Fig. 5:**
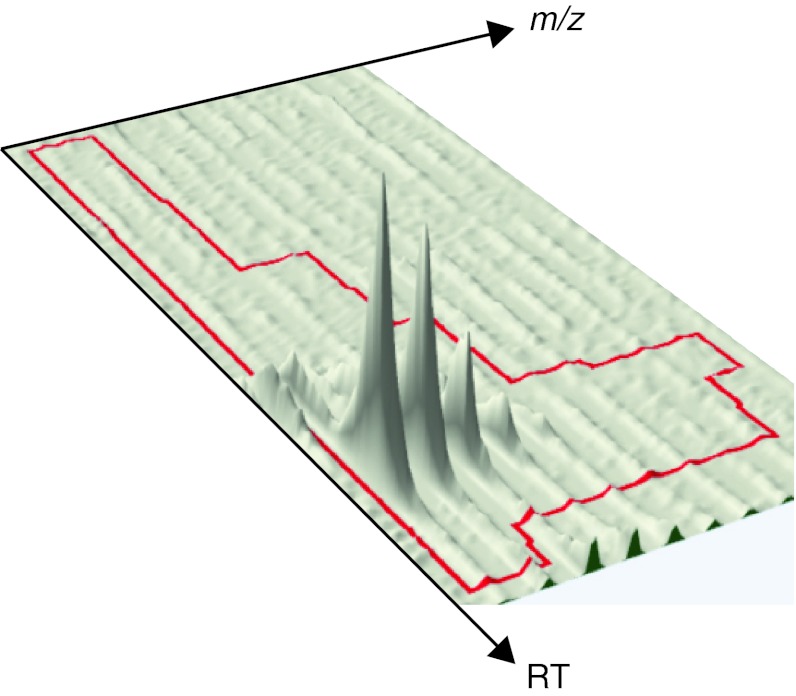
Incorrect feature detection. Chemical noise can mimic the isotopic distribution of a peptide signal and disturb *peak* detection algorithms

*Chemical noise* is mostly related to the detection of the LC mobile phase and buffers by the mass analyzer. It is more difficult to describe, as it behaves differently in the mass and time domains. In the mass domain, it has a periodic pattern very similar to that of the peptide signal, with which it often overlaps. In the chromatographic domain it appears as a slowly varying baseline, whose trend fluctuates over contiguous chromatograms according to the oscillation in mass. Figure 5 shows an example of incorrect feature detection in a Q-ToF dataset, caused by strong chemical noise mimicking the isotopic distribution of a peptide feature. In a previous work (Cappadona et al. 2008), we presented a novel signal model to disentangle all correlations between signal and noise and we proposed a method to access and remove both chemical and random noise through wavelet decomposition.

*Contaminants* can enter an MS experiment from a number of sources. Typical protein sources are the enzymes used in the sample preparation and contact proteins, such as keratins from skin cells. Although these proteins are usually defined as noise, their peptides actually elute and ionize exactly like the peptides under investigation. For this reason, their interference on feature detection has already been discussed in the previous section on isobaric co-eluting peptides.

**Fig. 6:**
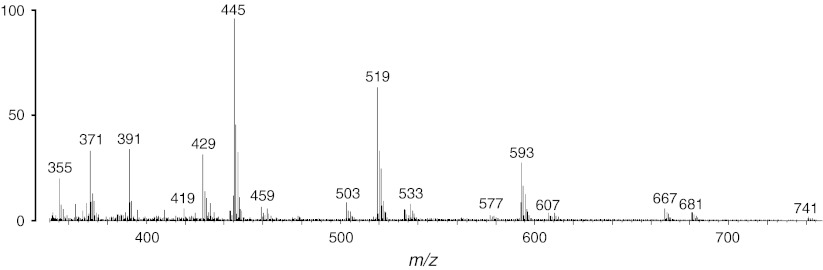
Typical contaminant *peaks*. An average of the first 10 min of the standard protein mix data set (Klimek et al. 2008), before the elution of any peptides. The accurate masses of the ions at *m*/*z* 429.1 and 445.1 are often used as lock mass calibrants (Olsen et al. 2005)

Nonprotein contaminants include plasticizers, surface contaminants and all kinds of chemicals normally present in the surrounding environment, such as perfumes and cleaning products. These long-term contaminants typically give singly charged signals and can be removed in a similar way to the chemical noise. In fact, their peaks are continuously dragged into the analyzer and therefore are not chromatographically resolved. Figure 6 shows some typical contaminant peaks in a shotgun proteomics experiment. The spectrum, which is an averaged MS survey scan of the first 10 min of an LCMS data set, before peptide elution, shows a very prominent set of polydimethylcyclosiloxane peaks, interfering with the real peptide signals.

Although advanced algorithms for feature detection have been presented in the literature, most quantification software tools still underestimate the importance of noise rejection. In some cases (e.g., Cox and Mann 2008), this step is neglected with the motivation that it is no longer necessary with high resolution mass spectrometers; in other cases (e.g., Khan et al. 2009), empirical thresholds are used to estimate detection and quantification limits, based on local signal to noise ratios (MacDougall and Crummett 1980). Figures 5 and 6 show that contaminant peaks and chemical noise, if not adequately removed, can cause either false positive or false-negative identifications by mimicking or masking the peptide signal. Software tools could still potentially investigate such cases by looking for unexplained peaks in MS/MS spectra or unexpected quantification ratios.

## Challenge 5: retention time alignment

Many of the issues pertinent to the quantification of labeled peptides also apply when analyzing label-free data; however, there exist additional challenges related to comparing peptide abundances across different LC–MS data files. The most important of these is that, although the *m*/*z* of a peptide can be determined with great precision by modern mass spectrometers, there can still be considerable variation in retention times, even between consecutive runs. This is still a significant problem despite the recent introduction of nano-LC systems without flow splitting and with computer controlled flow rates.

**Fig. 7:**
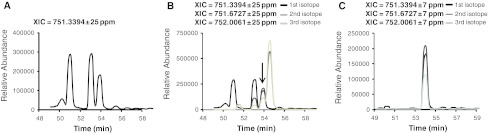
Improving *peak* detection in the presence of co-eluting peptides. XIC of the monoisotopic *peak* of the triply charged phosphopeptide IADPEHDHTGFLTEY(Phospho)VATR from the mouse mitogen-derived protein kinase Erk. **a** At 751.3394 Th ± 25 ppm at least 4 *peaks* co-eluted within a 5 min window, thus hampering *peak* detection. **b** XICs of the *second* and *third* isotopes allow identification of the only *peak*, marked with an *arrow*, for which the three isotopes perfectly co-eluted. **c** Specificity can also be increased by narrowing the mass window to 751.3394 Th ± 7 ppm. Analysis performed using Pescal (Cutillas and Vanhaesebroeck 2007)

The issue of retention time shifts has been addressed using alignment procedures (Finney et al. 2008; Vandenbogaert et al. 2008), which correct elution times by aligning them either to internal standards or to selected peaks in the total ion chromatogram (TIC) or the base peak chromatogram (BPC) of a reference run. The success of these procedures is strictly dependent on their ability to identify the same MS features across different runs. Matched features should then be aligned within pre-defined time and mass accuracy windows, which can be shifted by relative retention time approaches. Methods used for this purpose include dynamic time warping (DTW) and parametric time warping (PTW), along with their derivate algorithms (Christin et al. 2010; Finney et al. 2008). Narrow windows are usually chosen to decrease the probability of co-eluting isobaric compounds (Cutillas and Vanhaesebroeck 2007). Despite this expedient, the issue of co-eluting peptides cannot be totally avoided when dealing with complex mixtures and peak matching algorithms should be able to select the right peak for quantification. Figure 7 shows a case where different peptides co-eluted within a narrow time window. In this case, peak detection specificity can be improved by considering the charge and the theoretical isotope distribution of the peptide being quantified (Fig. 7b), in addition to the *m*/*z* and the retention time (Park et al. 2008). A further enhancement can be obtained by narrowing the mass window (Fig. 7c).

More recently, an algorithm has been published that performs the alignment based on MS/MS data (Tsou et al. 2010). This approach compares the retention times at which a peptide was fragmented and identified and uses linear regression to extrapolate information for runs lacking MS/MS data. Unfortunately, this strategy can only predict retention times to a certain degree, because MS/MS data are often triggered at peak tails, rather than at peak height, especially for very abundant peptides.

A shared feature of these peak alignment methods is that they rely on the occurrence of abundant peaks common to all samples. However, when comparing peptides from samples that are not closely related, the low number of common features might not allow confident peak alignment, as has been shown for primary tissues like cancer cells (Casado and Cutillas 2011). With these kinds of samples, in fact, the alignment algorithms can be disturbed by the presence of distinct peptides which have similar mass, but different retention times. In these cases, the introduction of internal standards that can be used as landmarks for alignment is highly recommended.

### Deuterium effect

Stable isotope labeling quantification is generally not affected by retention time shifts. The current consensus seems to be that deuterium is the only commonly used stable isotope that can be chromatographically resolved (Baldwin 2004; Zhang et al. 2001). For example, Hansen et al. (2003) demonstrated that, using reversed phase, heavy deuterium-containing peptides might elute several seconds prior to the corresponding light peptides. This so-called ‘isotope effect’ obviously complicates peptide matching, but has been shown to have little effect on the quantification accuracy, as long as the abundances of the deuterated peptides are measured along their whole elution profiles, rather than in one particular scan (Boersema et al. 2009; Ji and Li 2005). To overcome this issue, for instance, MSQuant allows for manual inspection of differentially expressed peptides and for proper integration over the entire XICs.

## Challenge 6: peptide identification

One of the main challenges in shotgun proteomics arises from incomplete data, because even the most advanced mass spectrometers cannot fragment all peptide ions present in the sample. In a recent paper, Michalski et al. (2011) showed that less than 20 % of the MS putative peptide features are targeted for fragmentation in data-dependent acquisition; and out of those, typically less than 60 % lead to identification. Therefore, despite being not directly related to the quantification process, peptide identification has a strong impact on the quantification rate. In principle, there are two ways to tackle this problem: trying to solve the poor fragmentation rate, or trying to increase the identification rate.

In data-dependent acquisition, the *fragmentation rate* is strictly instrument dependent and even the fastest instruments allow duty cycle rates of no more than 50 tandem spectra per second. In label-free quantification, this issue can be alleviated by means of exclusion lists, which instruct the instrument not to fragment peaks already identified in former runs. On a similar note, Smith et al. (2002) have proposed an accurate mass and time (AMT) tag approach, which relies on first establishing an AMT tag database for an organism, tissue or cell line, by performing high-resolution shotgun proteomic analysis, and then retrieving information from this database to obviate the need for subsequent MS/MS analyses.

The *identification rate* is also instrument dependent, in that high-resolution instrumentation has contributed to increase the rate from a few percent reported only few years ago (Mallick et al. 2007). Nevertheless, it is also dependent on the strategy used to infer peptide sequences from fragment mass spectra. These strategies can be broadly divided into three main categories (Nesvizhskii 2010): database searching, spectral library searching and de novo sequencing. Database searching is the most common approach and it is based on matching the observed spectrum to theoretical spectra generated from a protein sequence database. Library search methods generally outperform database search methods in terms of speed, error rates and sensitivity, but their applicability is contingent on the appropriate spectra being in the library (Lam and Aebersold 2011). Finally, de novo sequencing methods can be used for directly interpreting the acquired spectra, but they are computationally intensive and thus generally only used for unidentified high-quality spectra (Seidler et al. 2010). Given their complementary nature, some of these methods can be combined to increase the identification rate, as proposed for instance by the commercial package Peaks (Ma et al. 2003), which merges database search and de novo sequencing results. As quantification experiments often involve repeatedly running similar samples, then also using a package that can expand a spectral library based on database search identification could be beneficial.

### False discovery rates

Regardless of the identification method that is used, a very important aspect for protein quantification is the ability to estimate the number of incorrect identifications. In fact, retaining false-positive identifications in subsequent protein grouping can lead to incorrect protein ratios. The preferred method for calculating the false discovery rate is the target-decoy approach, originally proposed by Moore et al. (2002) and then extensively described by Elias and Gygi (2007) for database searches and by Lam et al. (2010) for library searches. This strategy is based on appending reversed, randomized or shuffled sequences to the original (‘target’) database before performing the search and then using these artificial (‘decoy’) sequences to evaluate the portion of false positive among all positive identifications. A false discovery rate (FDR) cutoff can then be set to limit the maximum number of accepted false-positive matches. Typical cutoff values range between 1 and 5 %, which means that a small portion of any identified peptides will be incorrect. If a large database is searched, these will typically be proteins with a single peptide hit, or ‘one-hit wonders’. However, if the database searched is small and the data set has a large number of spectra, it is also likely that they will come from the proteins with correct hits.

### Peptide modifications

Peptide modifications can seriously hamper protein identification and quantification and represent a major challenge in proteomic analysis. In fact, protein identification suffers from the combinatorial explosion of possible modification states, which increase exponentially with the number of modification sites (for example, a protein with three potential phosphorylation sites can be present in the sample in eight different states). At the same time, differential levels of amino acid modification between the samples can also seriously affect protein quantification, as each differently modified peptide should be quantified independently. In the case of post-translational modifications, these differential levels could be the purpose of the experiment, such as in phosphorylation monitoring (Gruhler et al. 2005; Iwai et al. 2010) but, in general, they can also be artifacts, reflecting unanticipated modifications related to sample preparation, rather than real changes in the relative abundance of the parent proteins.

Database search engines can be instructed to identify peptides with a set of specified modifications, including those introduced by sample handling and those present in vivo. In principle, any amino acid modification could be monitored and quantified by applying a range of mass shifts to all the residues in a peptide. It should be noted, though, that selecting a large number of variable modifications, by open or blind strategies (Chalkley et al. 2008; Tsur et al. 2005), could have a dramatic effect on the search speed and the false discovery rate. An effective strategy to overcome this problem may consist of running a first search, allowing few variable modifications against the full sequence database, followed by a second search, with a more complete set of modifications, but restricted to the proteins identified in the first round.

The assignment of PTMs, particularly phosphorylation, is never straightforward. Under collision-induced dissociation (CID) conditions the peptide is subjected to enough energy to cause loss of the phosphate moiety, observed as a neutral loss or even rearrangements of the phosphate groups. In turn, this neutral loss has the tendency to suppress sequence-diagnostic ion peaks, which makes assignment of the correct phosphorylation site very hard, particularly in case of multiple S, T or Y residues. Database search methods with site localization scoring have been designed specifically to extract additional information from the fragmentation spectra and to assign the correct position of the PTMs. These algorithms can be directly integrated into search engines, like the Mascot Delta Score (Savitski et al. 2011), embedded in Mascot, and the site localization in peptide (SLIP) scoring (Baker et al. 2011), embedded in the Batch-Tag search engine (Chalkley et al. 2008) of Protein Prospector. More often they require a particular search engine output for a second step of processing, as is the case for the H-Score (Savitski et al. 2010), the Ascore (Beausoleil et al. 2006) and the PTM score in MSQuant.

Alternatively, electron transfer dissociation (ETD) of a phosphorylated peptide has been proposed as a more reliable technique to obtain phosphosite localization, as it does not cause the neutral loss or rearrangement of the phosphate groups (Mischerikow et al. 2010).

Isotope labels are a particular case of peptide modification. Two different strategies are commonly adopted to identify labeled peptides. If the labeling state of peptide pairs is unknown, a single database search must be run, with the different tags set as variable modifications. If the labeling state is known beforehand, for instance because it has been determined by early feature detection, then separate database searches can be run, with each tag set as a fixed modification. This approach to customized database searches is often preferred, because it allows for smaller search spaces and better false discovery rates. It is also particularly necessary when the quantification method employed involves the labeling of multiple different residues, as in ^15^N quantification, where all peptides are labeled regardless of their amino acid composition, thus producing a variable mass shift between labeled pairs (Khan et al. 2011).

Library search methods can also be problematic for peptides with multiple modification sites, because it is unlikely that all the relevant permutations are present in the library.

## Challenge 7: normalization of peptide abundances

The result of feature detection and peptide identification is usually a table where each peptide is reported along with its own attributes, including mass, charge, retention time, modification state, proteins it might belong to and many more, depending on the software tool that performed the analysis. In the case of isotope labeling, the table will report the abundances of all isotopologues of a peptide, while in the case of a label-free experiment it will report the abundances in all the aligned runs in which a peptide was found. At this point, normalization of peptide abundances is essential for improvement of the quantitative accuracy of the experiment. In fact, changes in relative peptide abundances may reflect not just true biological differences but also systematic bias and random noise, resulting from sample preparation and instrumentation. Isotope labeling techniques are often preferred to label-free approaches because they reduce the perturbations related to sample handling. Nevertheless, data normalization is still required to account for variations in sample loading and for whenever multiple LC–MS runs are evaluated, for instance for comparison of multiple conditions. Normalization is therefore essential to reduce extraneous variability and to make abundances comparable both within and across samples. Many software tools automatically populate the table with normalized abundances and peptide ratios. Yet, these values might result from a correction strategy that does not fit the experimental setup, and a post-processing strategy based on the raw abundances could be a better option.

Ideally, extraneous variability can be addressed by normalizing to internal standards introduced early in the experimental workflow, as initially described for gel separated proteins and phosphorylated peptides (Cutillas et al. 2005) and later applied to bacterial proteins (Silva et al. 2006) and mouse tissues (Cutillas and Vanhaesebroeck 2007). However, normalization to internal standards may still not remove systematic bias arising as a consequence of differences in sample loadings. Thus, as an alternative, or in addition, peptide signals can be normalized by means of in silico procedures.

The most common approach for data normalization is based on the underlying assumption that only a small fraction of peptides is differentially expressed, while the majority should remain unchanged, and thus can be used for normalization. If peptide ratios deviate from unity, for instance due to errors in sample loading, a single normalization factor, based on the sum, average or median of all peptide abundances, can be used to minimize this offset. This technique is generally referred to as *global normalization*. Often, normalization values can also be obtained from a specific subset of features, for instance from spiked-in peptides used as internal standards, or a set of ‘house-keeping’ proteins assumed to be similarly abundant between samples. In these cases, the technique is referred to as *central tendency normalization.* This approach is particularly useful for datasets violating the basic hypothesis of equal expressions, for instance because sub-proteomes are differentially represented in the samples, or because samples are affected by nonsystematic contamination. In all cases, the set of features used for normalization should be carefully selected. Usually only peptides with abundances larger than a signal-to-noise threshold and common to all runs (or to a minimum percentage of runs) are retained. Modified peptides should also be filtered out, because their abundances might combine changes both in protein expression and in differential modifications (Wu et al. 2011). Scaling of abundances is also a common step, by which the distribution of peptide ratios is converted into a more symmetric, almost normal distribution. This is especially important if parametric tests, like the Student’s *t* test, will be used for differential analysis. When a logarithmic transformation is used to restore normality, data are usually plotted in MA (minus versus average) plots, showing the average log abundance on the *x* axis and the log fold change on the *y* axis. Such plots show the dependency of peptide ratios on the abundances from both samples, rather than just one, and allow for an easy observation of linear and nonlinear trends resulting from biases, which, in turn, can help choosing the best normalization strategy. The mentioned bias due to errors in sample loading, for example, usually results in the measured abundances of peptides from each sample being separated by a constant factor. In an MA plot this bias would show up as a constant deviation of peptide ratios from the *x* axis, which should be subtracted to center the plot and restore the hypothesis that most peptides are equally expressed. As already mentioned, in a simple case like this, the normalized abundance ratios can be calculated by subtracting the mean of the population of peptide ratios from the abundance ratio of each peptide. In the presence of outlier values, the median rather than the mean is often chosen as a more robust central value. Furthermore, when some ratio measurements are more reliable than others, it may be appropriate to weight the values in the calculation. For example, the program MaxQuant places the ratios into intensity bins, so that peptides with greater intensities are given more weight.

Other potentially more powerful normalization methods have been extensively benchmarked by Callister et al. (2006). If the systematic bias is not constant, but linearly dependent on the magnitude of the peptide abundances, *linear regression normalization* can be performed, by applying least square regression to the MA plot and by subtracting a proportionally larger amount of bias, estimated by the regression equation. Similarly, if the systematic bias is nonlinearly dependent on the magnitude of the peptide abundances, *local regression normalization* can be performed, by applying Lowess smoothing to the MA plot and by shifting the intensity-dependent Lowess line to 0. Finally, *quantile normalization* employs a nonparametric approach to restore similar peptide abundance distributions across samples. The conclusion of Callister’s study was that global normalization and linear regression ranked best in most cases. Similar conclusions were drawn by Kultima et al. (2009), who also found indications that the analysis order of the LC–MS experiments contributes to bias and developed a novel procedure, named RegRun, to improve linear regression by analysis order normalization. On a similar note, the recent Study 8 by the CPTAC network examined an extended pool of alternative sources for systematic bias, and regressed peptide ratios not only based on average abundance but also based on retention time, precursor *m*/*z*, peptide length, peptide length/z and mobile protons. The conclusion of the study was that intensity bias is the strongest when comparing samples analyzed by different labs, but RT bias is the strongest within labs (Rudnick et al. 2011).

## Challenge 8: protein inference

Except for peptidomics studies, peptide identification and quantification are just intermediate steps, an artifact of the bottom-up approach to proteomics. The meaningful analysis is at the protein level and the strategy chosen to rollup peptide identifications into protein identification is crucial for accurate quantification (Podwojski et al. 2010). The ‘protein inference problem’ has been described in several papers (e.g., Qeli and Ahrens 2010; Rappsilber and Mann 2002; Yang et al. 2004) and in a detailed tutorial by Nesvizhskii and Aebersold (2005). The main issue with protein inference is that it is an ill-posed problem, in that the mapping of peptides to precursor proteins is not always univocal. Shared peptides are peptide sequences that can be matched to more than one protein entry in a protein database and are more frequent than unique peptides, which can unequivocally be matched to a specific protein. Protein inference can thus be hampered by the presence of many causes of ambiguity. First of all, a single gene can result in hundreds of database entries, because of splicing variants, PTMs, protein isoforms and homologous proteins from other species. Furthermore, nonunique identifications may derive from truncated proteins, from similar domains in very different proteins, or from peptides that are short enough to occur randomly. Finally, multiple entries for the same protein can also occur in protein sequence databases due to sequencing or typographical errors. Discussions on how often this occurs can be found in Alexandridou et al. (2009) and Kohl et al. (2008).

Several software tools, including DTAselect (Tabb et al. 2002), ProteinProphet (Nesvizhskii et al. 2003) and IDPicker (Ma et al. 2009), automatically address the protein inference problem, by reporting all proteins with unique peptides and arranging the indistinguishable proteins into protein groups. The additional application of Occam’s razor results in a minimal list of proteins, accounting for all identified peptides. Early attempts to consider only unique peptides and to ignore the shared ones have been shown to under-represent the true amount of proteins and should be therefore avoided (Usaite et al. 2008).

MaxQuant creates protein groups if the set of identified peptides in one protein is equal to or completely contained in the set of identified proteins of another protein. For peptides that are shared between protein groups the number of peptides in each group is used as the assignment criterion. In the Matrix Science Mascot package (Perkins et al. 1999), protein groups with multiple members are subjected to hierarchical clustering, with the scores of nonshared peptide matches used as the distance metric (Koskinen et al. 2011). Dendrograms are then used to illustrate the relationship between family members and can be interactively cut to discard members judged to have insufficient evidence. Nesvizhskii and Aebersold (2005) have suggested that the quantitative information could be used to resolve some of the peptide grouping ambiguities.

Similar to peptides, proteins can also be incorrectly identified and FDR methods can be used to specify a proportion of false-positive identification matches. A minimum number of identified peptides per protein can be used as a criterion for reducing false-positive identifications (Carr et al. 2004), but this approach does not apply to small or low abundance proteins, which usually have less identifiable peptides. Manual identification of single-hits with information-rich peptides might thus help to reduce protein FDRs, while retaining valid single hits (Grobei et al. 2009).

## Challenge 9: protein quantification

Protein quantification is the final goal of many proteomics experiments. This task strictly relies on the correctness of all previously discussed steps, and especially on the outcome from peptide quantification and protein inference. Given a certain protein, two complementary methods have been proposed to rollup peptide quantification to protein quantification. The first consists of calculating different ratios from the protein’s peptides, followed by summarizing these ratios to obtain a single fold change. This method is commonly applied in stable isotopic labeling, but its use has been extended to label-free approaches (Old et al. 2005). Its main advantage is that a standard deviation of the protein ratio can be derived from the peptide ratios. The second method consists of deriving an estimate of the protein abundance from its peptides, followed by determining a single fold change at the protein level. In both cases, different metrics have been used to cluster peptides values around a central protein value. These metrics include sum, average, weighted average, median or any measure of central tendency. The sum is often used because it implicitly accounts for the decrease in measurement errors with larger intensities (Carrillo et al. 2010). Weighted average and median are usually preferred, because they are more robust, respectively, to the presence of low-quality measures and to outliers. In most cases, only a subset of peptides assigned to a given protein is used for quantification, because the main goal is to accurately determine the protein fold change, regardless of protein coverage, which has been already taken into account for protein inference. A common approach is to take the three most abundant peptides, based on the premise that the MS signals of the most efficiently ionized peptides directly correlate with the corresponding protein amount. This so-called Top 3 algorithm was originally proposed by Silva et al. (2006) for Q-ToF instruments running in LCMS^E^ mode, but has been validated recently also for ion trap-based mass spectrometers running in data-dependent acquisition (DDA) mode and compared to similar Top N approaches, which consider the N most abundant peptides (Grossmann et al. 2010).

The combination of two complementary methods, multiple summarization metrics and a further degree of freedom in selecting the appropriate high-quality peptides, gives rise to a whole plethora of possible quantification strategies. Comparing all strategies or suggesting a best one is beyond the scope of this article. For the purpose of this section we would rather point out that software developers should allow the user to explore various possibilities, while end-users should be aware of the influence of their choice on their final quantitative results.

### Protein quantification through spectral counting

An alternative approach for protein quantification makes direct use of ‘spectral counts’, the number of MS/MS identifications assigned to a protein. The rationale behind this method is that fragmentation events are proportional to protein abundance, although the linear range is strongly influenced by the settings for dynamic exclusion in data-dependent acquisition (Wang and Li 2008). Early analyses have used spectral counts as a semi-quantitative measure, to simply test differences in protein counts between different samples, until linearity has been confirmed over two orders of magnitude by comparison to spiked-in proteins in known concentrations (Liu et al. 2004).

### Absolute protein quantification

Since the empirical relationship with protein abundance has been proved, spectral counts have been used to calculate the absolute quantification of each protein within a mixture. Absolute concentration values are usually obtained by means of normalization procedures that correct for differing propensities of proteins to produce identifiable fragmentation spectra. These correction procedures range over a wide variety of techniques: NSAF, the normalized spectral abundance factor (Zybailov et al. 2006), simply divides counts by the protein length, analogously to the Fabb index (Aye et al. 2010), that normalizes by the protein molecular weight; emPAI, the exponentially modified protein abundance index (Ishihama et al. 2005), normalizes by the number of theoretically observable peptides; APEX, the absolute protein expression index (Lu et al. 2007), uses a machine-learning approach to derive prior expectation of observing each peptide.

Absolute protein copy numbers have recently been reported based on precursor ion currents (Schwanhäusser et al. 2011), rather than spectral counts. The technique, called intensity-based absolute quantification (iBAQ), proposes the sum of peak intensities of all peptides matching to a specific protein, normalized by the number of theoretically observable peptides, as an accurate proxy for protein levels.

## Challenge 10: statistical significance analysis and data mining

The ultimate goal of a quantitative proteomic experiment is often to compare protein expression levels between different groups. The data mining and functional interpretation of datasets to access biologically interpretable results pose many analytical challenges, which have been recently reviewed by Kumar and Mann (2009). Many quantitative software tools automatically output protein abundance ratios that can be used to discriminate regulated proteins, whose fold change exceeds a pre-defined, often arbitrary, threshold. However, they often lack proper algorithms for further statistical analysis, data mining and visualization, which are then usually ascertained by means of common statistical platforms, like the MATLAB Statistics Toolbox (The Mathworks Inc., Natick, MA) or the open source R statistical environment (R Development Core Team 2008); or by dedicated software packages, such as StatQuant (van Breukelen et al. 2009), DAnTE (Polpitiya et al. 2008) or the Perseus tool available with MaxQuant.

A statistical test is used to estimate a *p* value and a specified cut-off is chosen, such that below it protein changes are deemed significant. The testing procedure can then be evaluated by two common statistical measures, sensitivity and specificity, often conjunctly visualized by a receiver operating characteristic (ROC) curve. The most common statistical test used to evaluate differences between two groups is the two-sample *t* test. This test requires the assumptions of normally distributed data, easily checked by techniques such as the Shapiro–Wilk test. It also requires multiple samples to be present in each group, in order to estimate standard deviations. If the first hypothesis does not hold, as is generally the case for LC–MS abundances, which are restricted to positive values, log-transformation can be used to convert the observed abundance distribution into a more symmetric, almost normal distribution. Alternatively, nonparametric tests should be used, like permutation tests for the comparison of means, or the two-sample Kolmogorov–Smirnov test for the comparison of distributions. Nonparametric tests are especially useful when the sample size is low, since the data in this case often do not meet the normality assumption of the *t* test. If the second hypothesis does not hold, for instance because peptide ratios have already been combined to a single protein value, the one-sample *t* test should be used.

In many cases a proteomics experiment consists of many groups being compared. In this case, the analysis of variance (ANOVA) model can be chosen as a generalization of the *t* test, while the Kruskal–Wallis test can be used as a nonparametric alternative.

When multiple proteins are tested, the number of false-positive test results should be limited by a multiple testing correction. The Bonferroni correction, for instance, maintains the family-wise error rate under a desired significance level α by testing each of the *n* individual hypotheses at a significance level α/*n*. An alternative and less conservative approach is to adjust the *p* value to control the FDR. For this purpose, the *q* value has been introduced as a modified version of the *p* value that maximizes the number of true-positive statistical results, while controlling the proportion of false positives.

FDR procedures devised for the analysis of microarray data have also been tailored for the analysis of proteomics studies. For instance, Roxas and Li (2008) have demonstrated that the SAM method for significance analysis of microarrays (Tusher et al. 2001) can be effectively adapted to proteomics data for which, when compared to conventional *t* test, it provides richer information about protein differential expression profiles and better estimation of false discovery rates and miss rates. Similarly, Ting et al. (2009) have recently adopted LIMMA, linear models for microarray data (Smyth 2005), for normalization and statistical analysis of quantitative proteomics data, and they anticipate that more flexible frameworks for data analysis will become increasingly important for sophisticated experimental designs.

The major challenge for classification purposes is the high-dimensionality small-sample problem (Clarke et al. 2008), sometimes referred to as ‘large *p*, small *n*’, caused by the small number of samples available to mine a huge number of identified proteins. Also in this case, multivariate techniques devised in different contexts, like clustering and discriminant analysis, have been effectively adapted for proteomics purposes.

## Conclusions

A large number of technologies have emerged in the last decade for harvesting the quantitative information inherent in the mass spectrometry data from large-scale proteomics experiments. These frequently produce very large data sets, often consisting of thousands of MS and MS/MS spectra from hundreds of LC–MS runs. Software engineers, who write programs to process these data, as well as end-users, who wish to use these programs, need to be aware of the issues outlined in this paper, if they do not want to draw incorrect conclusions based on misleading results.

## Footnotes

1
http://www.taverna.org.uk/pages/wp-content/uploads/2011/06/Palmblad_ASMS_2011_LUMC.pdf.

2
http://www.thegpm.org/cRAP/index.html.
